# Interventions for nausea and vomiting in early pregnancy

**DOI:** 10.1590/S1516-31802011000100012

**Published:** 2011-01-06

## Abstract

**ABSTRACT:**

**BACKGROUND:**

Nausea, retching and vomiting are very commonly experienced by women in early pregnancy. There are considerable physical and psychological effects on women who experience these symptoms. This is an update of a review of interventions for nausea and vomiting in early pregnancy previously published in 2003.

**OBJECTIVES:**

To assess the effectiveness and safety of all interventions for nausea, vomiting and retching in early pregnancy, up to 20 weeks' gestation.

**SEARCH STRATEGY:**

We searched the Cochrane Pregnancy and Childbirth Group's Trials Register (28 May 2010).

**SELECTION CRITERIA:**

All randomized controlled trials of any intervention for nausea, vomiting and retching in early pregnancy. We excluded trials of interventions for hyperemesis gravidarum which are covered by another review. We also excluded quasi-randomized trials and trials using a crossover design.

**DATA COLLECTION AND ANALYSIS:**

Four review authors, in pairs, reviewed the eligibility of trials and independently evaluated the risk of bias and extracted the data for included trials.

**MAIN RESULTS:**

Twenty-seven trials, with 4041 women, met the inclusion criteria. These trials covered many interventions, including acupressure, acustimulation, acupuncture, ginger, vitamin B6 and several antiemetic drugs. We identified no studies of dietary or other lifestyle interventions. Evidence regarding the effectiveness of P6 acupressure, auricular (ear) acupressure and acustimulation of the P6 point was limited. Acupuncture (P6 or traditional) showed no significant benefit to women in pregnancy. The use of ginger products may be helpful to women, but the evidence of effectiveness was limited and not consistent. There was only limited evidence from trials to support the use of pharmacological agents including vitamin B6, and anti-emetic drugs to relieve mild or moderate nausea and vomiting. There was little information on maternal and fetal adverse outcomes and on psychological, social or economic outcomes. We were unable to pool findings from studies for most outcomes due to heterogeneity in study participants, interventions, comparison groups, and outcomes measured or reported. The methodological quality of the included studies was mixed.

**AUTHORS' CONCLUSIONS:**

Given the high prevalence of nausea and vomiting in early pregnancy, health professionals need to provide clear guidance to women, based on systematically reviewed evidence. There is a lack of high-quality evidence to support that advice. The difficulties in interpreting the results of the studies included in this review highlight the need for specific, consistent and clearly justified outcomes and approaches to measurement in research studies

**COMMENTS:**

Nausea and vomiting are common symptoms during pregnancy. Several therapies for these conditions have been described, including antiemetic drugs, vitamin B6, ginger, acupuncture, acupressure and acustimulation of the P6 point. The authors of this review assessed all the randomized controlled trials on any intervention to treat nausea, vomiting and retching during pregnancy.

The important conclusion from this review is that there is only limited evidence to support the use of pharmacological agents, including vitamin B6 and antiemetic drugs. Other interventions do not have any therapeutic value, based on the systematically reviewed evidence.
